# The NRF2 stimulating agent, tin protoporphyrin, activates protective cytokine pathways in healthy human subjects and in patients with chronic kidney disease

**DOI:** 10.14814/phy2.14566

**Published:** 2020-09-17

**Authors:** Richard A. Zager, Ali C. M. Johnson

**Affiliations:** ^1^ Department of Medicine University of Washington Seattle WA USA; ^2^ Fred Hutchinson Cancer Research Center Seattle WA USA

**Keywords:** acute kidney injury, heat shock, heme oxygenase 1, IL‐10, IL‐6, kidney, preconditioning, TGFβ1, tin protoporphyrin, TNF‐α

## Abstract

**Background:**

Tin protoporphyrin (SnPP), a heme oxygenase 1 (HO‐1) inhibitor, triggers adaptive tissue responses that confer potent protection against acute renal‐ and extra‐renal tissue injuries. This effect is mediated, in part, via SnPP‐induced activation of the cytoprotective Nrf2 pathway. However, it remains unclear as to whether SnPP can also upregulate humoral cytokine defenses, either in healthy human subjects or in patients with CKD. If so, then systemically derived cytokines could contribute SnPP‐induced tissue protection.

**Methods:**

SnPP (90 mg IV) was administered over 2 hr to six healthy human volunteers (HVs) and 12 subjects with stage 3–4 CKD. Plasma samples were obtained from baseline upto 72 hr post injection. Two representative anti‐inflammatory cytokines (IL‐10, TGFβ1), and a pro‐inflammatory cytokine (TNF‐α), were assayed. Because IL‐6 has been shown to induce tissue preconditioning, its plasma concentrations were also assessed. In complementary mouse experiments, SnPP effects on renal, splenic, and hepatic IL‐10, IL‐6, TGFβ1, and TNF‐α production (as gauged by their mRNAs) were tested. Tissue HO‐1 mRNA served as an Nrf2 activation marker.

**Results:**

SnPP induced marked (~5–7x) increases in plasma IL‐10 and IL‐6 concentrations within 24–48 hr, and to equal degrees in HVs and CKD patients. SnPP modestly raised plasma TGFβ1 without impacting plasma TNF‐α levels. In mouse experiments, SnPP did not affect IL‐6, IL‐10, TNF‐α, or TGFβ1 mRNAs *in kidney* despite marked renal Nrf2 activation. Conversely, SnPP increased splenic IL‐10 and hepatic IL‐6/TGFβ1 mRNA levels, suggesting these organs as sites of extra‐renal cytokine generation.

**Conclusions:**

SnPP can trigger cytoprotective cytokine production, most likely in extra‐renal tissues. With ready glomerular cytokine filtration, extra‐renal/renal “organ cross talk” can result. Thus, humoral factors seemingly can contribute to SnPP’s cytoprotective effects.

## Graphical abstract

Tin protoporphyrin can induce potent protective effects against organ injury. It does so by up‐regulating circulating and tissue factors that mitigate organ damage. This study demonstrates potential cytokine responses that can contribute to the agent's protective actions.

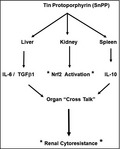

## 1 INTRODUCTION

In 1992, Nath et al. made the seminal observation that heme oxygenase 1 (HO‐1) plays a critical role in protecting kidneys against oxidant‐induced renal injury and associated AKI (Nath et al., 1992). He drew this conclusion by demonstrating that upregulation of intrarenal HO‐1 expression, induced via heme injection, conferred protection against the glycerol model of rhabdomyolysis‐induced AKI. The importance of HO‐1 in this protection was underscored by observations that the concomitant administration of the potent HO‐1 inhibitor, tin protoporphyrin (SnPP), exacerbated this AKI model. Following Nath's report, a plethora of studies, emanating from numerous laboratories, have confirmed that HO‐1 can confer protection against diverse forms of renal, as well as extra‐renal, injuries (e.g., Facchinetti, 2020; Nath, 2006).

Despite overwhelming evidence that HO‐1 can exert cytoprotective effects, there is a seemingly contradictory literature indicating that within 18–24 hr of SnPP administration, adaptive tissue responses arise which usher in profound tissue protection against superimposed injuries (e.g., ref. Atef, El‐Fayoumi, Abdel‐Mottaleb, & Mahmoud, 2017; Cukiernik, Mukherjee, Downey, & Chakabarti, 2003; Johnson, Delrow, & Zager, 2017; Johnson & Zager, 2018; Juncos et al., 2006; Kaizu et al., 2003; Panizzon, Dwyer, Nishimura, & Wallis, 1996; Sutherland et al., 2011; Uchida et al., 2003; Zager, 2015; Zager, Johnson, & Frostad, 2016). For example, our laboratory has reported that within 18 hr of SnPP injection, mice develop marked protection against diverse forms of ischemic and toxin‐induced AKI (Johnson et al., 2017; Johnson & Zager, 2018; Zager, 2015; Zager et al., 2016). This delayed SnPP “preconditioning” response has similarly been reported in extra‐renal tissues (e.g., brain, lung, liver, heart, lung, and retina; ref. 4–9). Based on our prior studies, we have advanced the following mechanistic hypothesis for this SnPP‐induced cytoprotective state (Johnson et al., 2017; Johnson & Zager, 2018): *first*, SnPP evokes transient (~4 hr) oxidative stress, as evidenced by acute reductions in renal glutathione and increases in protein carbonyl and malondialdehyde content; *second*, this transient oxidative stress activates the cytoprotective Nrf2 pathway (increased Nrf2 binding to nuclear antioxidant response elements; AREs); *third*, the resulting Nrf2 activation triggers broad based increases in diverse anti‐oxidant and anti‐inflammatory gene transcription (e.g., HO‐1, SRXN1, GCLC); and *fourth*, the resulting increases in diverse cytoprotective proteins within kidney evokes resistance to superimposed ischemic and toxin‐mediated renal damage. The importance of the Nrf2 pathway to SnPP‐mediated renal protection is underscored by the absence of this response in Nrf2 (−/−) deficient mice (Johnson & Zager, 2018).

In addition to being the so called “master regulator” of anti‐oxidant defenses, Nrf2 can also exert potent anti‐inflammatory effects (e.g. ref. Jesus, Araujo, Zhang, & Yin, 2012; Paine, Eiz‐Vesper, Blasczyk, & Immenschuh, 2010; Wardyn, Ponsford, & Sanderson, 2015). This has led us to question whether SnPP might also induce anti‐inflammatory responses, potentially within the cytokine cascade. IL‐10 is the quintessential immunosuppressive cytokine, evoking its action via diverse cellular and humoral pathways (Paine et al., 2010). Following its generation and release from monocytes, CD4+ Th2 cells and B cells, IL‐10 binds to a 110 kDa high affinity receptor. After engagement, suppression of a host of pro‐inflammatory pathway results (e.g., decreases in pro‐inflammatory cytokine production; suppressed NF‐κB activity; reduced HLA‐class II and CD14 cell surface expression) (Gabryšová, Howes, Saraiva, & O'Garra, 2014; Opal & DePalo, 2000; Schottelius, Mayo, Sartor, & Baldwin, 1999). The importance of IL‐10 as a modulator of AKI has been well documented. For example, administration of exogenous IL‐10 or IL‐10 inhibitor(s) have been shown to mitigate, or exacerbate AKI, respectively (Akai et al., 2019; Andres‐Hernando et al., 2017; Soranno et al., 2016; Tadagavadi & Reeves, 2010). In this regard, Faubel et al. have advanced the following hypothesis (Andres‐Hernando et al., 2017; Soranno et al., 2016): (a) AKI causes renal IL‐6 synthesis; (b) renal release of IL‐6 stimulates splenic immune cell IL‐10 production; and (c) with splenic IL‐10 release, an “organ cross talk” loop is completed as spleen‐derived IL‐10 inhibits AKI‐triggered inflammation/AKI severity.

Given the above considerations, the present study was undertaken to ascertain whether SnPP administration can trigger protective cytokine production. To this end, both healthy human volunteers and subjects with advanced (stage 3–4) CKD were administered SnPP, followed by measurements of plasma IL‐10, IL‐6, as well as anti‐inflammatory TGFβ1 levels (Opal & DePalo, 2000). To determine whether a potentially countervailing pro‐inflammatory response might also occur, plasma TNF‐α levels were assessed. Finally, to determine potential direct effects of SnPP on renal and extra‐renal IL‐6, IL‐10, TGFβ1, and TNF‐α production, their mRNAs were measured in renal, splenic, and hepatic tissues obtained from SnPP‐treated and control mice. The results of these complementary clinical and experimental studies form the basis of this report.

## 2 METHODS

### 2.1 Subject recruitment

Both healthy volunteers (*n*, 6) and patients with either stage 3 or stage 4 CKD (*n*, 6 per group) formed the basis of the current study (Zager, Johnson, Guillem, Keyser, & Singh, 2020). These subjects were previously reported in a prior study (Zager et al., 2020) which assessed the effects of SnPP on Nrf2 dependent antioxidant gene expression (heme oxygenase 1, NAD(P)H dehydrogenase [quinone] 1, p21, and ferritin). Stored plasma samples from that study were used to generate the currently reported data. Participant eGFRs were calculated using the CKD Epidemiology Collaboration formula where CKD3 and CKD4 are defined by eGFR ranges of 30–59 ml/min per 1.73 m^2^ (CKD3) and 15–29 ml/min per 1.73 m^2^ (CKD4), respectively. All subjects were residents of Central Florida. Institutional Review Board (IRB) approval was issued by Advarra IRB, Columbia, MD. Informed consent was obtained from each study participant. The Fred Hutchinson Cancer Research Center (FHCRC) waived IRB approval for laboratory testing because only analyses of de‐identified plasma samples were conducted at this site. The study was conducted in adherence with the Declaration of Helsinki. Inclusion criteria for the healthy volunteers (HVs) included male and female subjects aged 18–80 years, a body weight < 125 kg, and the absence of any acute or chronic disease or chronic drug administration. Female subjects were required to have had a negative pregnancy test or be post‐menopausal, post‐tubal ligation, or regularly use effective contraception. Inclusion criteria for the participants with CKD included ages of 18–80 years and body weight < 125 Kg. Study exclusion criteria included pregnancy, and any significant medical illness other than CKD or diabetes. All subjects must have been able to comply with all study procedures. This study was enrolled in Clinicaltrials.gov prior to subject recruitment (NCT0363002; NCT03893799).

### 2.2 Clinical protocol

Each of the participants received a 2‐hr intravenous infusion of 90 mg SnPP (Cascade Custom Chemistry, Portland, OR) in 100 ml of saline. Heparinized plasma samples were collected prior to drug infusion (baseline; 0 hr), and 4, 8, 12, 24, 48, and 72 hr post infusion and stored at −70°C. Following the infusion, the participants remained overnight at the study site (Riverside Clinical Research, Edgewater, FL) to screen for potential adverse events. In addition, potential delayed adverse events were assessed for 28 days post infusion at the study site, and included routine hematology, hepatic/cardiac enzymes, and EKG monitoring. Potential adverse renal effects were assessed by serial measurements of BUN, plasma creatinine, urinary albumin/creatinine ratios, and urinary AKI biomarkers (NGAL, KIM‐1, cystatin C, and N acetyl‐glucosaminidase) (Zager et al., 2020). A four‐member data safety monitoring board (DSMB) reviewed all of these safety data and confirmed no adverse changes in these parameters.

Plasma IL‐10 values were measured by Halifax Laboratory Services, Daytona Beach, FL. Plasma IL‐6, TGFβ1, and TNF‐α were measured at FHCRC. To this end, frozen plasma samples were shipped overnight on dry ice from the study site to FHCRC. Upon receipt, the samples were thawed and aliquoted into 96‐well plates for subsequent ELISA testing. IL‐6, TGFβ1, and TNF‐α were determined with commercially available ELISA kits (IL‐6: DY‐206; TGFβ1: DY‐240; TNF‐α: DY‐210; all kits from R & D Systems, Minneapolis, MN). Each sample was assayed in duplicate, with values calculated from “within plate” standard curves. Standards were provided by the kit manufacturer.

### 2.3 Plasma SnPP concentrations

To determine SnPP pharmacokinetics, plasma samples were obtained from six normal subjects at 1, 2, 4, 6, 8, 12, 18, and 24 hr following SnPP injection. They were processed by protein precipitation (releasing protein bound SnPP), followed by supernatant analysis by HPLC with fluorescence detection (performed by MicroConstants, Inc, San Diego, CA). To assess the potential impact of CKD on plasma SnPP disappearance rates, the above assessment was completed in six subjects with stage 4 CKD. Peak SnPP concentrations, plasma SnPP half‐lives, and areas under the curve (AUC; geometric means) were determined and compared between the two groups.

### 2.4 SnPP effects on mouse kidney and spleen IL‐10, IL‐6, TGFβ1, and TNF‐α gene expression

The following study was undertaken to assess the direct impact of SnPP on kidney, splenic, and hepatic IL‐10, IL‐6, TGFβ1, and TNF‐α production, using mice to provide surrogate tissue samples for humans. To this end, six male CD‐1 mice (30–40 g; from Charles River Laboratories; Wilmington, MA) received a tail vein injection (100 µl) of 0.75 mg SnPP [approximately 1.5 × the equivalent human dose of 90 mg/70 Kg; based on an FDA conversion factor of 12:1]. Six vehicle injected mice served as controls. Four hours post injection, the mice were deeply anesthetized with pentobarbital (40–50 mg/kg), the abdominal cavity was opened and then the left kidney, spleen, and a liver lobe were resected. The tissues were iced, and total RNA was extracted (RNeasy kit; Qiagen, Germantown, MD). For kidney analysis, cortical tissues were obtained. The primary target mRNAs in each organ included IL‐6, IL‐10, TGFβ1 and TNF‐α mRNA. However, in order to gain a broader assessment of potential SnPP‐induced renal cytokine changes, MCP‐1 mRNA was also assessed. The mRNA values were determined by RT‐PCR and factored by simultaneously assessed GAPDH levels (Zager et al., 2016). As a positive control, and to serve as an index of Nrf2 activation, HO‐1 mRNA was assessed. Heat shock protein 70 mRNA responses were also determined to dissociate HO‐1 (aka HSP‐32), changes from a generic heat shock protein response.

### 2.5 Calculations and statistics

The primary clinical outcomes were changes in plasma IL‐10, IL‐6, TGFβ1, and TNF‐α levels over time following SnPP infusion. The data were analyzed by ANOVA for repeated measures. Secondary clinical outcomes were potential differences in baseline plasma IL‐10, IL‐6, TGFβ1, and TNF‐α concentrations between the HV and the CKD subjects (analyses by unpaired two sided Student's *t* test). Because no significant differences in cytokine levels were observed between CKD3 and CKD4 subjects, the data from these two groups were combined, forming a single CKD group for ease of data presentation. All clinical values within the text are given as means ± 1 *SD*. Because of wide inter‐subject variations in plasma IL‐6 and TNF‐α concentrations, these values were converted to log base10 prior to depiction and statistical analyses. Variance is depicted in the figures as 95% confidence intervals.

Mouse renal cortical, splenic, and hepatic mRNA values are presented as means ± 1 *SEM*. Comparisons between control and SnPP‐treated groups were made by unpaired Student's *t* test. Statistical significance throughout the studies was judged by a p value of < 0.05.

## 3 RESULTS

### 3.1 Study participant demographics and safety monitoring

Age, gender, body weight, blood pressure, prevalence of diabetes, and eGFRs for the study groups are presented in Table 1. The eGFR ranges for the CKD 3 and CKD 4 groups were 44–57 ml/min per 1.73 m^2^ and 15–25 ml/min per 1.73 m^2^, respectively. As previously reported (Zager et al., 2020), no demonstrable adverse renal effects were induced by SnPP administration [unchanged eGFRs, BUNs, creatinine, urine albumin/creatinine ratios, or urinary biomarker (KIM‐1, NGAL, NAG, or cystatin C) concentrations]. Mild photosensitivity was observed in approximately 15% of patients, as previously reported (Zager et al., 2020).

**TABLE 1:** Baseline demographics in study subjects

Criterion	Healthy volunteers	CKD−3 (30–59 ml/min/1.7 ± 3 m^2^)	CKD4 (15–29 ml/min/1.73 m^2^)
Age	54 (7)	73 (7)	69 (5)
Gender (M/*F*%)	50/50%	67/33%	83/13%
% White race	50%	67%	67%
Weight (Kg)	85 (14)	78 (19)	100 (17)
Diabetes	0%	42%	66%
BP systolic/diastolic	128/22 (21/8)	139/79 (14/7)	132/76 (18/9)
eGFR (ml/min)	87 (4)	51 (5)	21 (4)

Note

Mean values and 1 *SD* (in parentheses) are presented.

### 3.2 Plasma IL‐10 concentrations

**FIGURE 1:**
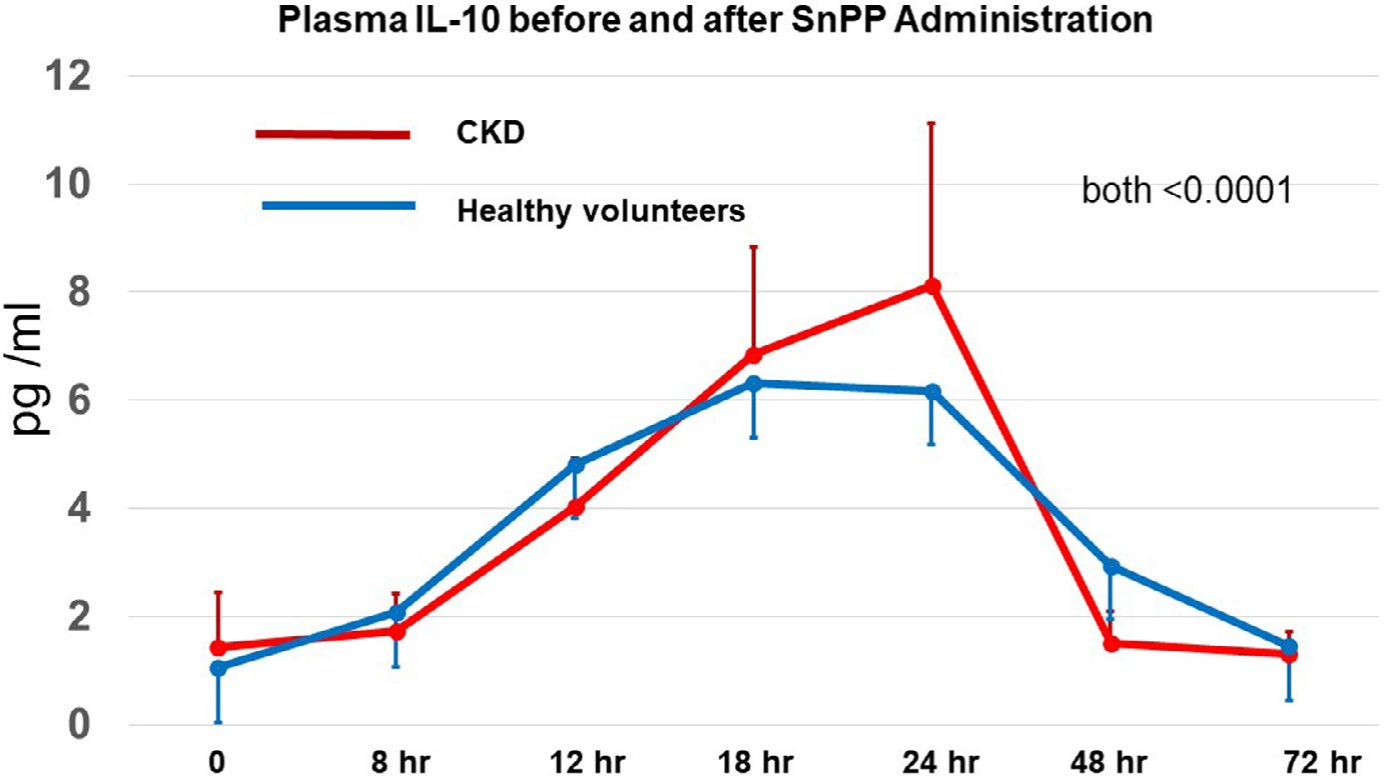
SnPP administration increases plasma IL‐10 concentrations in healthy volunteers and subjects with CKD. Within 4–8 hr of receiving 90 mg of SnPP, plasma IL‐10 increases were observed, reaching peak values (~5–6x baseline) within 18–24 hr. Sharp plasma IL‐10 decrements were observed thereafter, returning to baseline levels within 48–72 hr. No significant differences in responses were noted between the healthy volunteers and CKD group. *p* values for both groups were <0.0005 as determined by ANOVA for repeated measures

Baseline plasma IL‐10 concentrations did not significantly differ between the HV (1.44 ± 0.96 pg/ml) and the combined CKD group (1.05 ± 1.35 pg/ml; *p* = .47). As shown in Figure 1, both groups showed comparable and progressive IL‐10 increases after SnPP injection, starting within 8 hr and reaching peak values between 18 and 24 hr (*p* < .0001; ANOVA, repeated measures). The peak values were approximately four‐ to fivefold higher versus baseline. Subsequently, plasma IL‐10 levels promptly fell, returning to baseline within 48–72 hr.

### 3.3 Plasma IL‐6 concentrations

**FIGURE 2:**
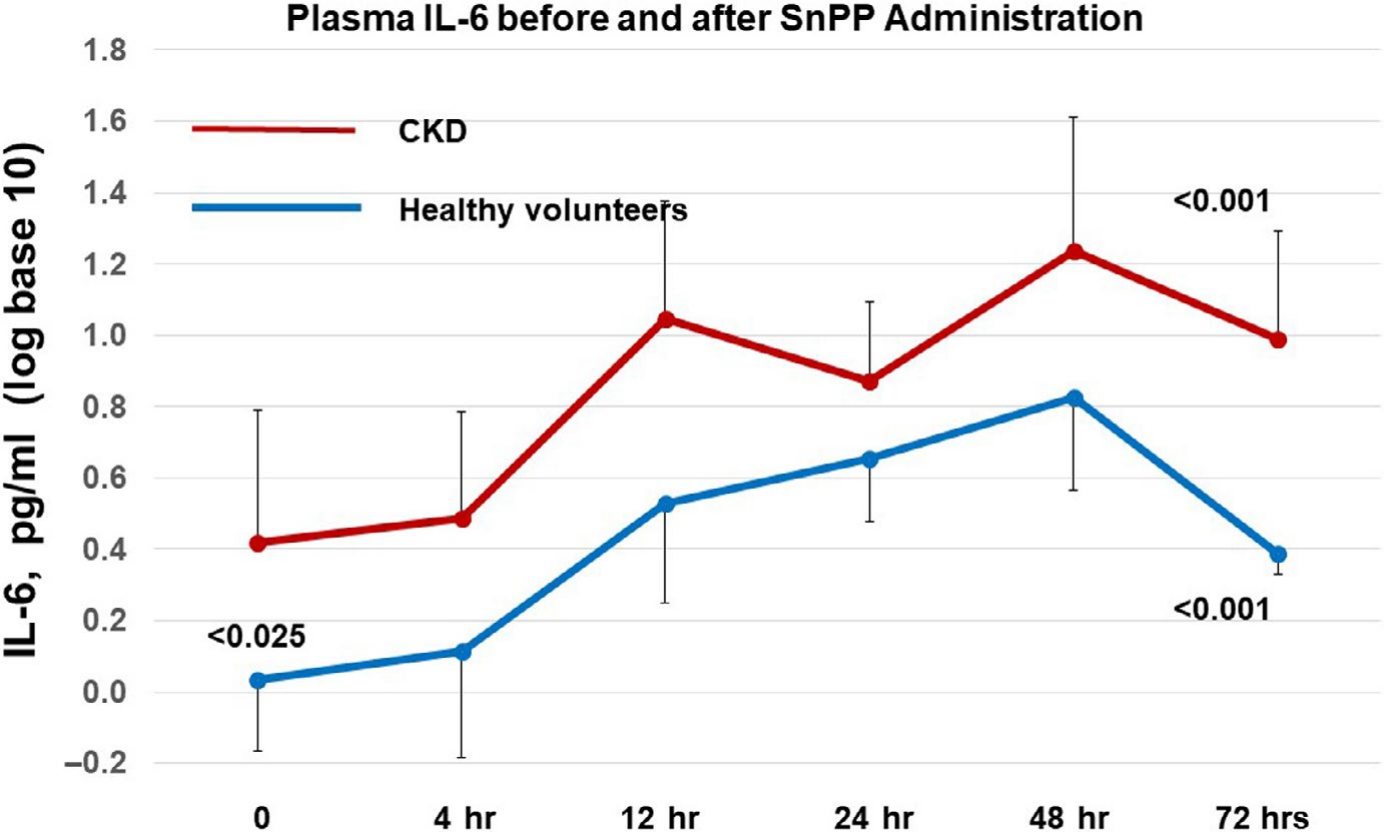
SnPP administration increases plasma IL‐6 concentrations in healthy volunteers and subjects with CKD. Baseline plasma IL‐6 levels were significantly higher at baseline in the CKD versus the healthy volunteer group (<0.025). Despite the differing baseline IL‐6 concentrations, the fold increase over baseline values did not significantly differ for the CKD (7.25‐fold increase) versus the HV group (6.9‐fold increase). By 72 hr, plasma IL‐6 declines were apparent, but had still not returned to baseline levels. The values are presented after log base 10 transformation

Due to a high degree of variance, plasma IL‐6 values were analyzed after conversion to log base 10. At baseline, the IL‐6 values were significantly higher in the CKD (0.42 ± 0.51) versus the HV group (0.04 ± 0.51; pg/ml; *p* < .025; see Figure 2). Both groups demonstrated statistically significant increases in plasma IL‐6 concentrations over time, reaching peak values at 48 hr post SnPP injection (*p* < .001 by ANVOA, repeated measures). Despite the differing baseline IL‐6 concentrations, the fold increase over baseline values did not significantly differ for the CKD (7.25‐fold increase) versus the HV group (6.9‐fold increase). By 72 hr, plasma only modest IL‐6 declines were apparent.

### 3.4 Plasma TGFβ1 concentrations

**FIGURE 3:**
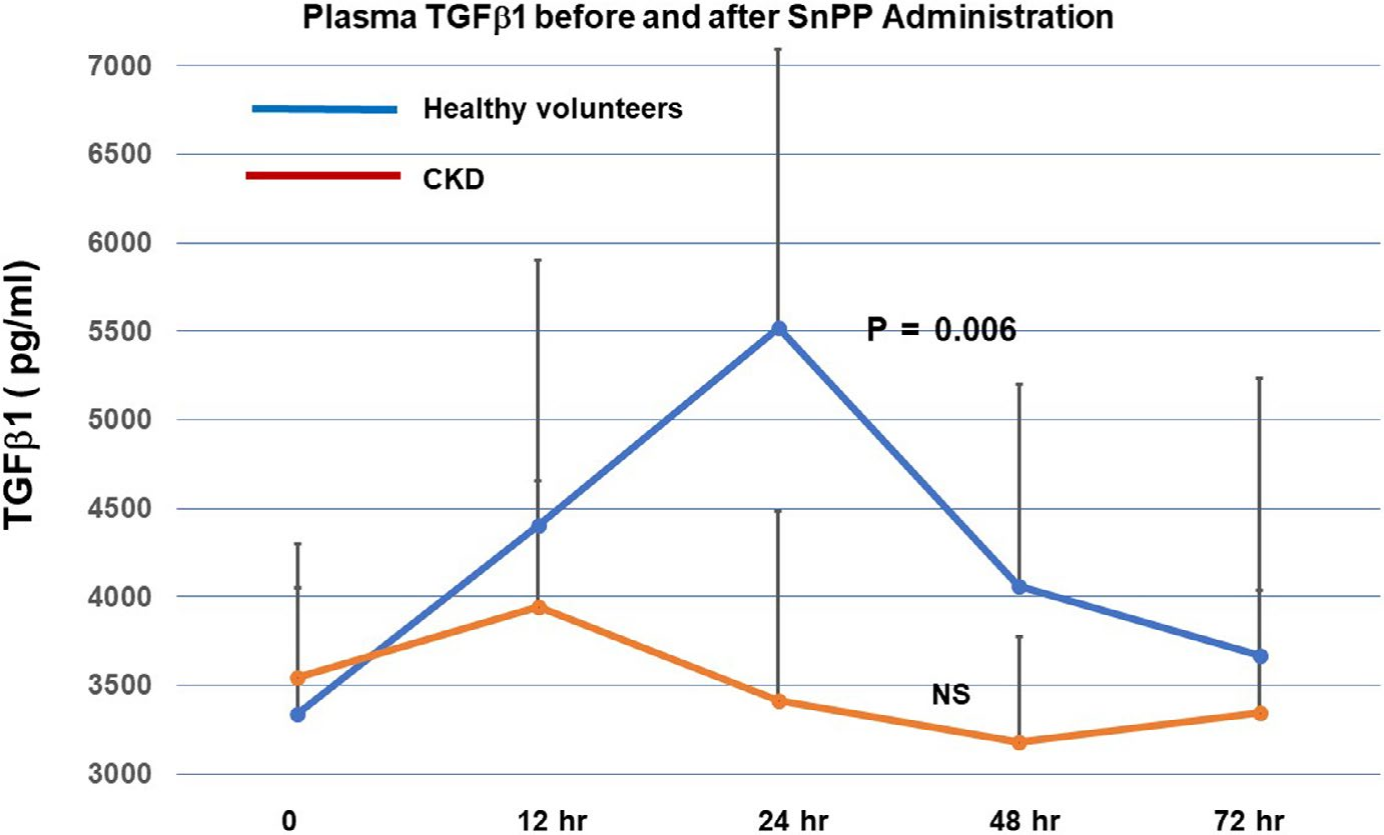
SnPP administration increases plasma TGFβ1 concentrations in healthy volunteers, but not in CKD subjects. Baseline TGFβ1 levels did not differ between the healthy volunteer and the CKD groups. SnPP administration induced an approximate 75% increase in plasma TGFβ1 levels in healthy subjects whereas no response was observed in the CKD cohort

Baseline plasma TGFβ1 concentrations were nearly identical in the HV and CKD subjects (Figure 3). In the HV group, SnPP caused an approximate 75% increase in TGFβ1 concentrations within 24 hr. This was followed by steep declines thereafter. In contrast, the CKD subjects failed to manifest a SnPP‐induced TGFβ1 response.

### 3.5 Plasma TNF‐α concentrations

**FIGURE 4:**
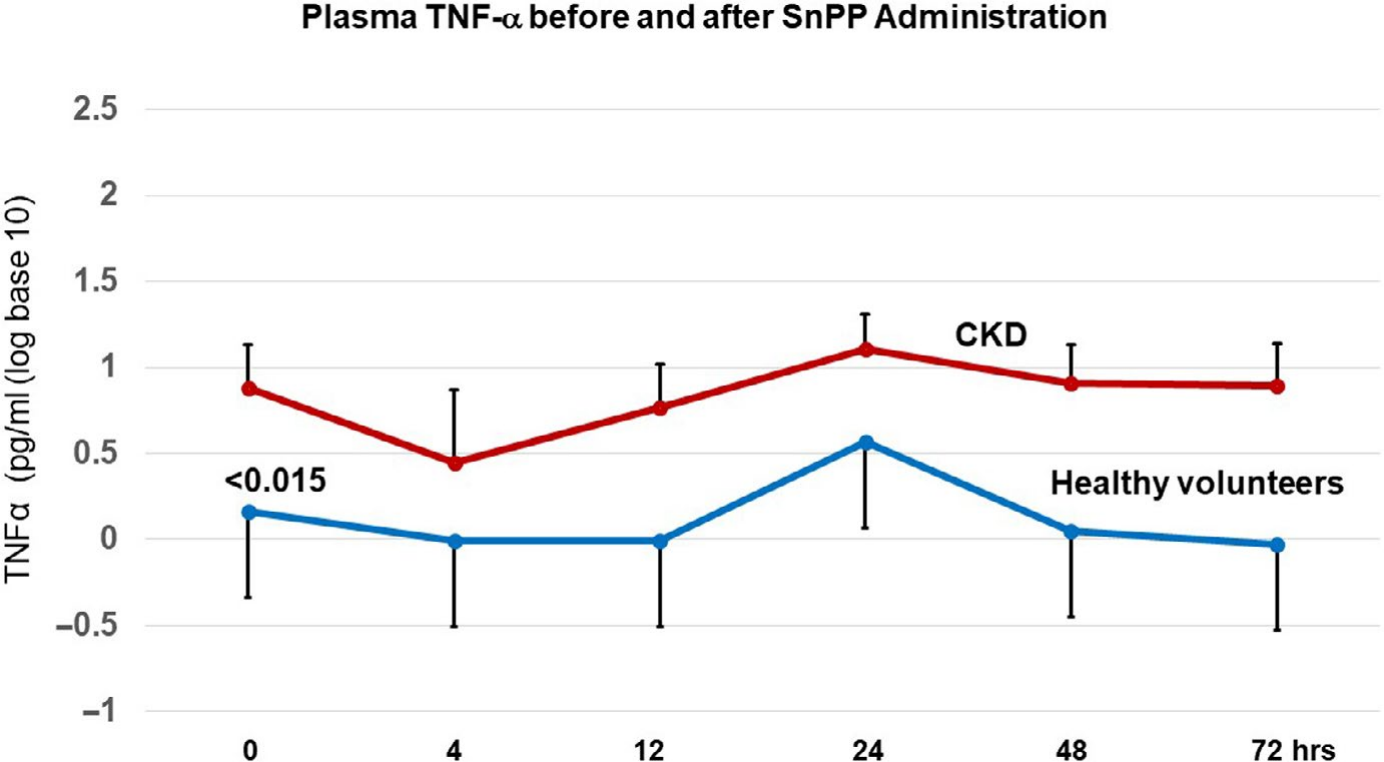
Plasma TNF‐α concentrations were elevated in CKD subjects versus healthy volunteers at baseline, although neither group demonstrated material TNF‐α changes in response to SnPP administration. Baseline TNF‐α levels were approximately sixfold higher in CKD subjects versus healthy volunteers. However, neither group manifested substantial TNF‐α changes in response to SnPP administration. The values are presented after log base 10 transformation

Baseline TNF‐α concentrations were approximately sixfold higher in the CKD versus the healthy volunteer group (Figure 4; *p* < .015). Following SnPP injection, the plasma TNF‐α values remained relatively stable throughout 72 hr of observation. Reflective of their higher baseline values, the post SnPP concentrations remained consistently higher over time in the CKD versus the HV group.

### 3.6 SnPP plasma pharmacokinetics

**FIGURE 5:**
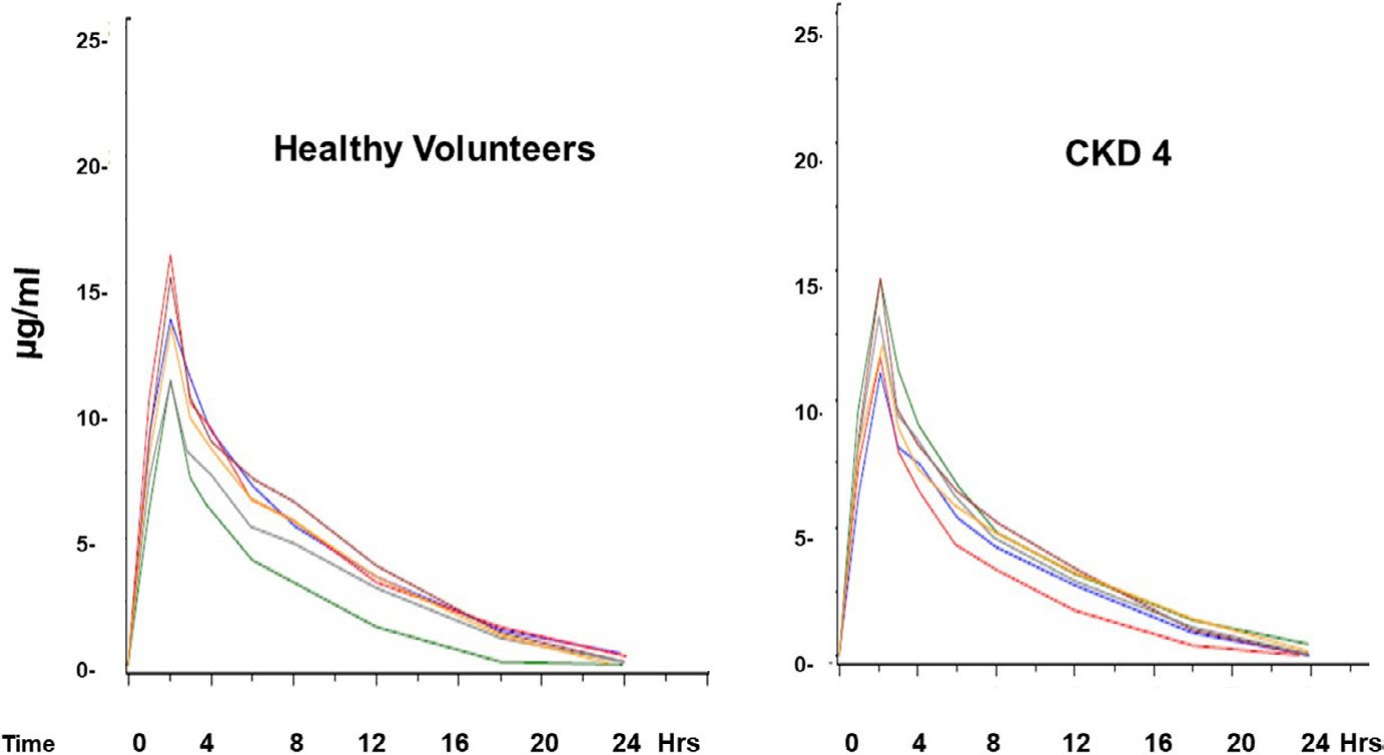
Following SnPP injection, exponential declines in SnPP plasma concentrations occurred. The graphs show “spaghetti plots” of values observed in the six healthy volunteers and six subjects with stage 4 CKD. Despite the marked differences in eGFRs, no significant differences in peak SnPP concentrations, SnPP half‐lives (3.5 hr), or AUCs for the two groups were observed

Individual plasma SnPP concentrations for each healthy volunteer and each CKD4 participant are depicted in Figure 5. An exponential decline in plasma SnPP values was observed in each subject. The peak (Nath et al., 1992) SnPP plasma concentrations (~15 μg/ml), and the plasma SnPP half‐lives (~3.5 hr) did not differ between the healthy volunteers and CKD4 subjects. The lack of impact of CKD on plasma SnPP disappearance was also evidenced by highly comparable AUCs for the two groups (HVs, 88 ± 13; CKD4, 94 ± 19; µg × hr/ml; mean ± 1 *SD*).

### 3.7 Mouse renal cortical cytokine mRNA assessments

**FIGURE 6:**
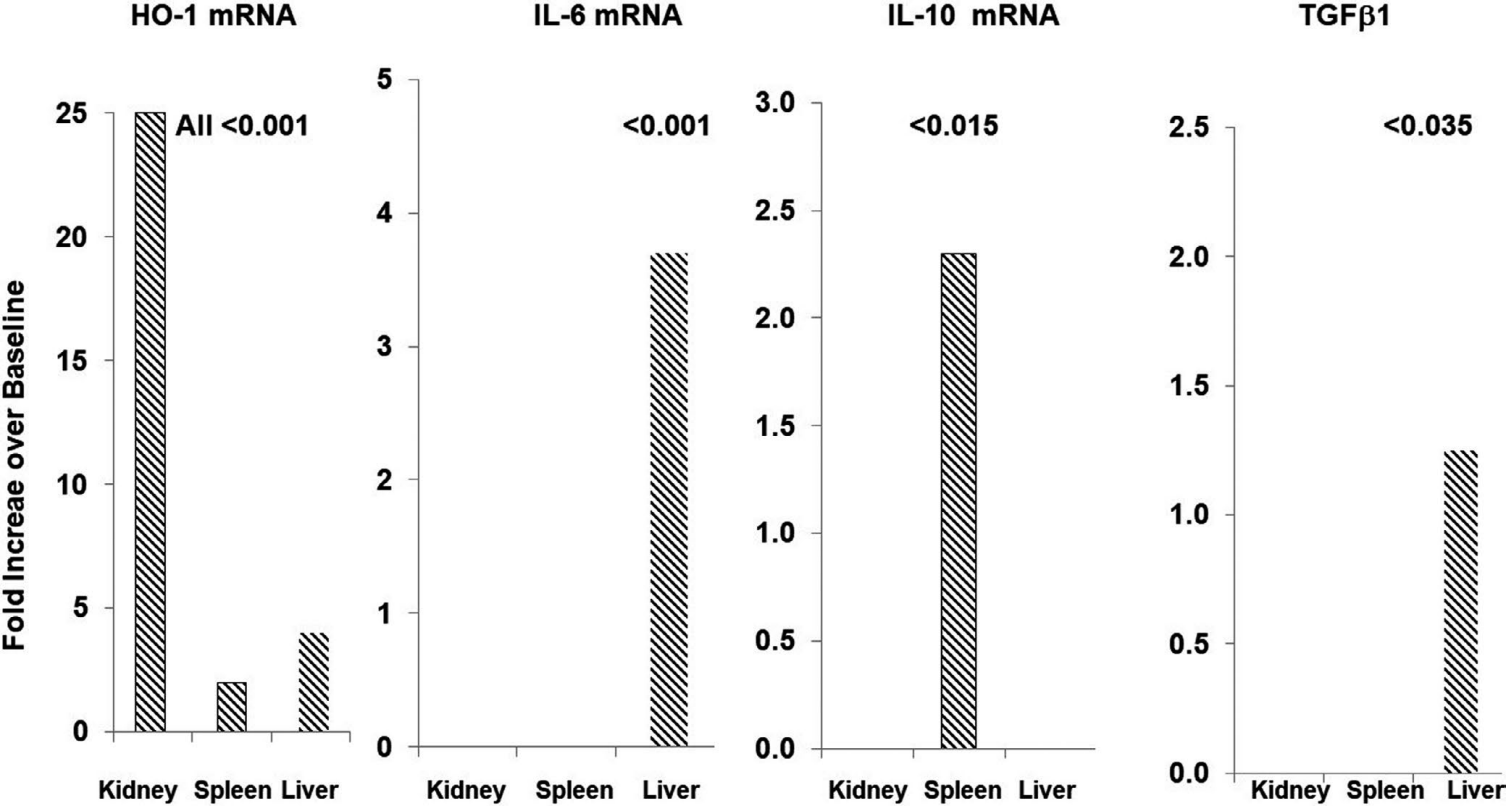
SnPP‐induced fold increases in HO‐1, IL‐6, IL‐10, and TGFβ1 mRNA levels in mouse tissues at 4 hr after SnPP administration. SnPP induced significant increases in HO‐1 mRNA in kidney, liver, and spleen. However, IL‐10 mRNA increases were confined to spleen. Only the liver responded with increases in IL‐6 and TGFβ1 mRNA concentrations. The values represent the mean fold increases over baseline values. Mean values ± *SEM* are presented in Tables 2, 3, 4

As shown in Table 2, and Figure 6, SnPP induced a 25‐fold increase in renal cortical HO‐1 mRNA within 4 hr of its injection, confirming prior observations of Nrf2 activation (Johnson et al., 2017; Johnson & Zager, 2018; Zager, 2015; Zager et al., 2016). Although HO‐1 is a heat shock protein (i.e., HSP‐32), the lack of a corresponding HSP‐70 mRNA elevation indicated that the HO‐1 mRNA elevation did not reflect a non‐specific heat shock protein response. SnPP had no effect on intrarenal IL‐10, TGFβ1, or IL‐6 mRNAs, suggesting that the plasma IL‐10, TGFβ1, and IL‐6 elevations observed in the human subjects likely did not result from renal IL‐6, IL‐10, or TGFβ1 generation. Noteworthy was that SnPP also did not raise renal TNF‐α or MCP‐1 mRNAs. Thus, these findings support prior conclusions of SnPP renal safety studies (Zager et al., 2020), given that no changes in these pro‐inflammatory cytokine mRNAs were observed.

**TABLE 2:** Mouse renal cortical mRNA levels 4 hr following SnPP or vehicle administration

Analyte mRNA	Vehicle	SnPP (4 hr)	*p* value
**HO−1**	0.05 ± 0.0	1.42 ± 0.38	<0.0001
HSP−70	1.64 ± 0.47	1.90 ± 0.74	NS
IL−10	0.49 ± 0.14	0.72 ± 0.24	NS
IL−6	0.28 ± 0.12	0.32 ± 0.13	NS
TNF‐α	0.12 ± 0.02	0.15 ± 0.05	NS
MCP−1	0.64 ± 0.07	0.60 ± 0.05	NS
TGFβ1	0.62 ± 0.02	0.58 ± 0.04	NS

Note

Results are presented as ratios to simultaneously determined GAPDH product. SnPP activity was confirm by a 25‐fold increase in HO‐1 mRNA. This was not due to a non‐specific heat shock protein response, given the lack of increase in HSP70 mRNA. SnPP did not activate any of the five tested cytokine/chemokine genes. Values are means (±1 *SEM*).

### 3.8 Mouse splenic cytokine mRNA assessments

As presented in Table 3 and Figure 6, SnPP caused a 2.3‐fold increase in splenic IL‐10 mRNA, implying resident splenic immune cell IL‐10 generation. However, no other changes in splenic cytokine gene expression was observed. Splenic Nrf2 activation was implied by a doubling of splenic HO‐1 mRNA.

**TABLE 3:** Mouse spleen mRNA levels 4 hr following SnPP or vehicle injection

Analyte mRNA	Vehicle	SnPP (4 hr)	*p* value
HO−1	1.1 ± 0.1	1.8 ± 0.1	<0.005
HSP−70	2.4 ± 0.4	2.0 ± 0.3	NS
IL−10	0.70 ± 0.10	1.62 ± 0.3	<0.015
IL−6	1.0 ± 0.2	1.3 ± 0.3	NS
TGFβ1	2.4 ± 0.2	2.3 ± 0.2	NS

Note

SnPP activated the HO‐1, but not the HP70 gene. It also caused 2.3‐fold increase in IL‐10 mRNA. However, the remaining cytokine mRNAs remained at control levels. Values are means (±1 *SEM*). Results are presented as ratios to simultaneously determined GAPDH product.

### 3.9 Mouse hepatic cytokine assessments

SnPP induced a three‐ to fourfold increase in hepatic IL‐6 mRNA levels (Table 4). A far more modest, but still significant, increase (~25%) in hepatic TGFβ1 mRNA was also observed. As with kidney and spleen, SnPP also increased hepatic HO‐1 imRNA without an HSP‐70 response.

**TABLE 4:** Mouse liver mRNA levels 4 hr following SnPP or vehicle injection

Analyte mRNA	Vehicle	SnPP (4 hr)	*p* value
HO−1	0.13 (0.01)	0.61 (0.06)	<0.0001
HSP−70	0.75 (0.1)	0.95 (0.1)	NS
IL−10	0.52 (0.14)	0.65 (0.12)	NS
TGFβ1	2.4 (0.1)	3.0 (0.2)	<0.035
IL−6	0.3 (0.1)	1.1 (0.1)	<0.001

Note

SnPP activated the HO‐1, but not the HSP‐70 gene. It also caused a near fourfold increase in IL‐6 mRNA. A modest, but significant, increase in TGFβ1 mRNA was also observed. Values are means (± 1 *SEM*). Results are presented as ratios to simultaneously determined GAPDH product.

## 4 DISCUSSION

A highly complex and intricate network of immune responses can impact both the initiation and propagation phases of tissue injury. Prominent among these is a pleomorphic group of pro‐ and anti‐inflammatory cytokines/chemokines and their receptors. Rather than a single cytokine being the prime determinant of cytokine‐mediated tissue injury, it appears more likely that the ratio of pro‐inflammatory versus anti‐inflammatory cytokines is determinative. For example, low IL‐10/TNF‐α ratios, rather than absolute plasma TNF‐α or IL‐10 concentrations, best correlated with adverse outcomes in patients with acute respiratory distress syndrome (ARDS) (Goodman et al., 2001). Stenvinkel et al. reached a similar conclusion in studies of uremia‐induced inflammation, where low IL‐10/TNF‐α ratios corresponded with accelerated cardiovascular disease and the “uremic wasting” syndrome (Stenvinkel et al., 2005).

Although Nrf2 activation within renal tissues appears to be the dominant pathway through which SnPP induces its cytoprotective effects (Johnson et al., 2017; Zager et al., 2016), whether these changes are associated with altered systemic cytokine profiles has not been defined. A recent study from our laboratory documented that SnPP activates the Nrf2 pathway in both healthy human subjects and patients with advanced CKD (Zager et al., 2020). We have now sought to determine whether this SnPP‐Nrf2 effect is associated with changes in pro versus anti‐inflammatory cytokine profiles.

Towards this end, we first selected IL‐10 for study because it is widely believed to be the most potent of the anti‐inflammatory cytokine defenses (Opal & DePalo, 2000). Indeed, within 18–24 hr, five‐ to sevenfold plasma IL‐10 elevations were observed. Of note, this is the same time frame at which the height of the SnPP‐induced cytoprotective state is expressed (Johnson et al., 2017; Zager et al., 2016). Of interest, the presence of CKD did not impact the SnPP‐induced plasma IL‐10 increases. Given its small size (18 kDa), IL‐10 undergoes ready glomerular filtration and urinary excretion. Hence, the presence of advanced CKD might be expected to decrease renal IL‐10 clearance, and thus, further support plasma IL‐10 increases. Finally, it is important to note that a strong correlation exists between plasma and renal cortical IL‐10 protein concentrations in SnPP‐treated mice (Zager et al., 2016). This clearly implies that the presently observed SnPP‐induced plasma IL‐10 elevations in humans were associated with increased intrarenal IL‐10 concentrations. Of interest, SnPP only increased IL‐10 mRNA expression within splenic tissues. This suggests that resident splenic (and possibly circulating) T cells and B cells were the sources of the SnPP‐triggered the five‐ to sevenfold plasma IL‐10 increases.

Although IL‐6 has long been considered to be a pro‐inflammatory cytokine, it also exerts potent anti‐inflammatory effects (Billah et al., 2019, 2020; Filho et al., 2015; Kimizuka et al., 2004; McGinnis et al., 2015; Nechemia‐Arbely et al., 2008). As examples, IL‐6 can suppress production of pro‐inflammatory cytokines and their receptors. It also stimulates glucocorticoid (Bethin, Vogt, & Muglia, 2000) and IL‐10 production (Andres‐Hernando et al., 2017; Soranno et al., 2016). Exogenous IL‐6 administration has been reported to mitigate inflammation during ischemic‐reperfusion injury (Billah et al., 2019; Kimizuka et al., 2004). Furthermore, endogenous IL‐6 has been implicated as a mediator of remote‐ as well as of direct‐ischemic preconditioning (Billah et al., 2019; Kimizuka et al., 2004; Nechemia‐Arbely et al., 2008). In light of these considerations, we sought to determine whether SnPP‐induced preconditioning induces an IL‐6 response. Indeed, this was the case, with five‐ to sevenfold plasma IL‐6 elevations being observed in both HVs and CKD subjects within 24–48 hr of SnPP administration. Whether these IL‐6 increases triggered splenic IL‐10 production, as suggested by Faubel et al. (Andres‐Hernando et al., 2017; Soranno et al., 2016), remains unknown. However, in stark contrast to Faubel's findings in which AKI evoked *renal* IL‐6 increases, in the case of SnPP, only *hepatic* IL‐6 mRNA increases were observed. This suggests that the liver, and not the kidney, was the likely source of the SnPP‐mediated plasma IL‐6 elevations.

Because TGFβ1, like IL‐10, is considered to be an anti‐inflammatory cytokine, its concentrations post SnPP injection were also assessed. Notably, an approximate 75% increase in TGFβ1 levels was observed in healthy volunteers within 24 hr. However, the CKD subjects failed to demonstrate this SnPP ‐ TGFβ1 response. It is noteworthy that baseline TGFβ1 levels were nearly identical in the CKD and HV cohorts. This implies that CKD is not associated with an overall suppression of the TGFβ1 pathway. Hence, the reason for the absent SNPP‐ TGFβ1 response in CKD subjects remains unknown. In mice, SnPP induced a modest increase in hepatic TGFβ1 mRNA, suggesting liver as a potential site for increased TGFβ1 production. However, the relatively small mRNA response (25% increase) suggests that other sites of TGFβ1 generation (e.g., circulating immune cells directly exposed to SnPP) may well have been in play.

To gauge whether SnPP might exert a countervailing pro‐inflammatory response, plasma TNF‐α levels were measured at both baseline and following SnPP injection. Baseline TNF‐α levels were markedly elevated in the CKD patients versus normal subjects, consistent with CKD being a pro‐inflammatory state (Stenvinkel et al., 2005). However, following SnPP injection, no substantive TNF‐α increases were observed. Given the SnPP‐induced plasma IL‐10 elevations, and absent TNF‐α changes, an approximate six‐ to sevenfold increase in IL‐10/TNFα ratios resulted. As previously noted, high IL‐10/TNFα ratios have been associated with an “anti‐inflammatory state” (e.g., in patients with ARDS or uremia; 26,27). If so, then this ratio could serve as a “biomarker” of a SnPP‐induced cytoprotective state.

It is well recognized that the presence of CKD represents a major risk factor for the development of AKI, for example, during cardiopulmonary bypass surgery. Thus, if one were to deploy SnPP preconditioning for AKI prevention in such patients it would be critical to know whether SnPP pharmacokinetics are altered by renal insufficiency. The results of this study suggest the answer is no, given the finding of virtually identical SnPP half‐lives (~3.5 hr) and plasma disappearance curves in healthy volunteers and stage 4 CKD subjects. Despite its small size (750 daltons), SnPP is tightly bound to hemopexin and albumin which greatly retards glomerular filtration (Anderson, Simionatto, Drummond, & Kappas, 1984). Hence, rapid tissue uptake, for example, in liver (Anderson et al., 1984), rather than renal clearance, appears to be the prime determinant of SnPP’s rapid plasma disappearance rate.

The above studies leave a number of important and unanswered questions. *First*, we cannot conclude as to whether the observed SnPP‐induced plasma IL‐6, TGFβ1, and IL‐10 increases are secondary results of Nrf2 activation, or whether they arise from a direct SnPP drug effect. To answer this question, future studies could test whether SnPP increases plasma IL‐6, IL‐10, and TGFβ1 levels in Nrf2 ‐/‐ mice. *Second*, although there is an extensive literature which indicates that IL‐10, TGFβ1, and IL‐6 can each exert anti‐inflammatory and cytoprotective actions, it remains to be proven that their plasma elevations mechanistically contribute to SnPP’s overall preconditioning effect. To resolve this issue, degrees of SnPP‐mediated protection might be tested in IL‐6, IL‐10, or TGFβ1 deficient mice. *Third*, while the liver and spleen respond to SnPP with IL‐6/TGFβ1 and IL‐10 mRNA elevations, respectively, it remains quite possible that additional organs or cell types might also contribute to the observed plasma cytokine increases. Finally, although we have attempted to define sites of SnPP‐induced cytokine production in mice, the relevance of these results to the human tissue responses remains unknown. Hence, the above considerations suggest areas for future investigation.

**FIGURE 7:**
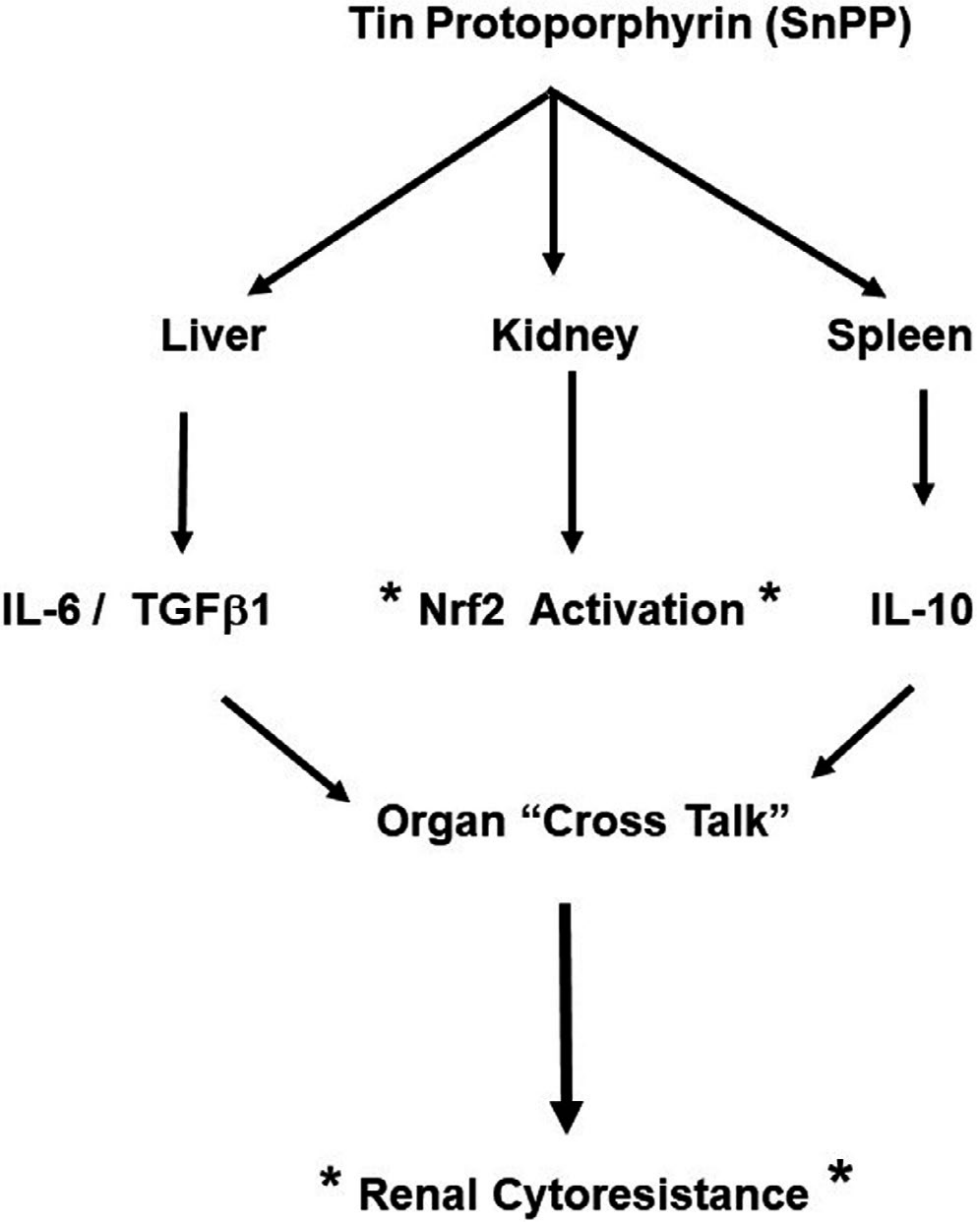
Depiction of potential humoral mechanisms that may contribute to SnPP‐mediated cytoresistance. In addition to activating the Nrf2 pathway (denoted by asterisks) SnPP activates IL‐10 in spleen and IL‐6/TGF beta 1 in liver (as assessed by their mRNAs). Elevations in their plasma concentrations leads to renal filtration and “organ cross talk”. This allows for these cytoprotective cytokines to contribute to Nrf2’s cytoprotective activities

In conclusion, this study provides the first evidence that SnPP can activate the IL‐6, IL‐10, and TGFβ1 cytokine pathways in humans, as evidenced by marked increases in their plasma concentrations with 18–48 hr of SnPP injection. Based on the present mouse experimental data, extra‐renal sites, most notably spleen and liver, appear to be major sources for these cytokine increases. Given their small sizes, (≤18 kDa) these three cytokines undergo ready glomerular filtration, thereby allowing tubular cell access and the induction of previously documented kidney protective effects. Thus, organ “cross talk” phenomena, in addition to direct SnPP‐driven Nrf2 tissue activation, may each contribute to SnPP‐mediated induction of a renal preconditioning state, as depicted in Figure 7.

## AUTHOR CONTRIBUTIONS

Dr. Zager designed the study, interpreted the data and wrote the manuscript. Ms Johnson designed and performed all assays and analyzed all data. She also edited the final manuscript and made important contributions to it.

## ETHICAL STATEMENT

Dr. Zager is a paid consultant to Renibus Therapeutics. The study was funded in part by Renibus Therapeutics and in part by discretionary research funds from the Fred Hutchison Cancer Research Center.
